# Advanced cognitive impairment among older nursing home residents

**DOI:** 10.1186/s12877-021-02336-1

**Published:** 2021-06-23

**Authors:** Tadeja Gracner, Patricia W. Stone, Mansi Agarwal, Mark Sorbero, Susan L Mitchell, Andrew W. Dick

**Affiliations:** 1 RAND Corporation, 1776 Main Street, Santa Monica, CA 90401 USA; 2 Center for Health Policy, Columbia University School of Nursing, 560 W. 168th St, New York, NY 10032 USA; 3 Washington University School of Medicine, 660 S Euclid Ave, St.Louis, MO 63110 USA; 4 RAND Corporation, 4570 Fifth Ave #600, Pittsburgh, PA 15213 USA; 5 Hebrew Senior Life Marcus Institute for Aging Research, Boston, MA USA; 6 Department of Medicine, Beth Israel Deaconess Medical Center, Boston, MA USA; 7 RAND Corporation, 20 Park Plaza #920, Boston, MA 02116 USA

**Keywords:** Advanced cognitive impairment, End-of-Life, Alzheimer’s disease, Dementia, Survival

## Abstract

**Background:**

Though work has been done studying nursing home (NH) residents with either advanced Alzheimer’s disease (AD) *or* Alzheimer’s disease related dementia (ADRD), none have distinguished between them; even though their clinical features affecting survival are different. In this study, we compared mortality risk factors and survival between NH residents with advanced AD and those with advanced ADRD.

**Methods:**

This is a retrospective observational study, in which we examined a sample of 34,493 U.S. NH residents aged 65 and over in the Minimum Data Set (2011–2013). Incident assessment of advanced disease was defined as the first MDS assessment with severe cognitive impairment (Cognitive Functional Score equals to 4) and diagnoses of AD or ADRD. Demographics, functional limitations, and comorbidities were evaluated as mortality risk factors using Cox models. Survival was characterized with Kaplan-Maier functions.

**Results:**

Of those with advanced cognitive impairment, 35 % had AD and 65 % ADRD. At the incident assessment of advanced disease, those with AD had better health compared to those with ADRD. Mortality risk factors were similar between groups (shortness of breath, difficulties eating, substantial weight-loss, diabetes mellitus, heart failure, chronic obstructive pulmonary disease, and pneumonia; all *p* < 0.01). However, stroke and difficulty with transfer (for women) were significant mortality risk factors only for those with advanced AD. Urinary tract infection, and hypertension (for women) only were mortality risk factors for those with advanced ADRD. Median survival was significantly shorter for the advanced ADRD group (194 days) compared to the advanced AD group (300 days).

**Conclusions:**

There were distinct mortality and survival patterns of NH residents with advanced AD and ADRD. This may help with care planning decisions regarding therapeutic and palliative care.

**Supplementary Information:**

The online version contains supplementary material available at 10.1186/s12877-021-02336-1.

## Background

Alzheimer’s disease (AD) and related dementias (ADRD) are the 6th leading cause of death in the United States (US) [1–3]. Unless another fatal illness intervenes, these diagnoses will progress to an advanced stage, ultimately resulting in death [4]. Approximately 70 % of persons with AD or ADRD die in nursing homes (NHs) [5]. Accurately identifying a health state we refer to as advanced disease is therefore critical to providing optimal care; as is differentiating mortality risk factors and survival patterns across groups because underlying causes of decline are heterogeneous.

Although AD is the most recognized form of dementia, its clinical features and cognitive impairments affecting survival are different from ADRD, which includes dementia with Lewy bodies (DLB), frontotemporal dementia, Parkinson’s, mixed dementia, as well as vascular or multi-infarct dementia [6–8]. Researchers have found that ADRD patients have shorter survival compared to those with AD [9–11], that their survival depends on sex, cognitive level, neuropathology [2, 3, 12–14], and that AD and ADRD patients differ in their co-existing functional impairments or co-morbidities [10, 15, 16]. For instance, eating difficulties and related weight-loss are common complications in patients with AD, and are a significant predictor of end of life [14, 17]. On the other hand, for patients with ADRD such as DLB, walking and fall-related injuries are among the largest mortality risks [18, 19], whereas these risks are not as substantial for patients with AD. For some other types of ADRD, such as vascular dementia, hypertension, diabetes, and stroke remain the most important mortality risk factors [20, 21]. Finally, evidence shows that disease progression is more rapid for those with ADRD than for patients with AD [22, 23].

However, the previous work studying AD and especially ADRD does not focus on its most severe and advanced stage. Though work has been done studying survival and mortality risks in people with *either* advanced AD *or* ADRD [3, 11–13, 18, 19, 24], less is known about whether residents with advanced AD differ from those with advanced ADRD in their mortality risk factors and survival patterns [14, 25, 26]. Lack of prognosticators across AD and ADRD may thus fail to recognize dementia as a terminal illness for these patients.

Early identification of when NH residents with either AD or ADRD enter the stage of advanced cognitive impairment may improve end of life care. Though previous researchers have used the routinely collected Minimum Data Set (MDS) to identify residents with advanced cognitive impairment [27], none have distinguished between AD and ADRD [28]. Using routine MDS assessments, our goal was to identify incident advanced cognitive impairment as observed in MDS and evaluate the differences in survival and mortality risk factors for those with advanced AD and those with advanced ADRD.

## Methods

This is a retrospective cohort study of a nationally representative sample of US NH residents with advanced AD or ADRD between 2011 and 2013. The Institutional Review Boards approved this study.

### Data source

We used data from the 2011 to 2013 MDS 3.0, which is a comprehensive, standardized resident screening and assessment tool, federally mandated for use in all licensed US NHs. We included the routine assessments for NH residents that are required upon admission (or readmission), quarterly thereafter, and at any time there is a significant change in health status. We also included assessments that are completed at or around 5, 14, 30, 60, and 90 days following admission or readmission from hospital. We obtained Vital Status data linked to individual MDS identifiers, providing date of death for each NH resident through November 2016.

### Study population and measures

We started with a random sample of 1 million NH residents aged 65 and above who had at least one quarterly or annual assessment from 2011 to 2013. From these, we identified residents for whom we observed the incident advance cognitive impairment assessment in MDS, defined as the first assessment on which the criteria were satisfied, with at least one prior quarterly, annual, or admission assessment in which they were not satisfied. These criteria required a diagnosis of AD (MDS item I4200) and/or a diagnosis of ADRD. For ADRD, we use the MDS item I4800, which refers to Non-Alzheimer’s dementia, including DLB, frontotemporal dementia, Parkinson’s or Creutzfeldt-Jakob disease, mixed dementia, or vascular or multi-infarct dementia. In addition, a cognitive function score (CFS) equal to 4 (i.e., severely impaired) had to be present on the same assessment [29, 30]. We did not use ICD9 for any diagnoses. How CFS is obtained has been explained elsewhere [30].

In our data, most of the NH residents who were diagnosed with dementia had only ADRD diagnoses (65 %), but a significant fraction had both ADRD and AD diagnoses (21 %), and only 14 % had AD diagoses only. To explore how different or similar their survival patterns were, we estimated survival and hazard functions for each of these three mutually exclusive groups. Residents with AD and those with AD and ADRD exhibited similar survival patterns, but different patterns from those who were diagnosed only with ADRD (see the Supplement, eFigure 1). Guided by these empirical patterns, we divided our sample into two mutually exclusive groups: those with AD (with or without an ADRD diagnosis) and those with ADRD (without an AD diagnosis), hereafter referred to as AD and ADRD, respectively. Like others, we assumed that AD and ADRD were considered permanent once diagnosed [14, 29]. To protect against data errors and false positives, we required advanced cognitive impairment to be present on at least two assessments unless there were only two or fewer assessments remaining before death (in which case we required only one positive assessment).

### Predictors of survival

Based on the literature, demographics, functional status, comorbidities, and other health conditions were selected a priori as potential predictors of survival [2, 10, 14–16, 18, 19, 26, 31–33]. Residents’ demographic characteristics included age (60–74, 75–84, 85–94, and 95+), sex, marital status (yes/no), and race/ethnicity (American Indian or Alaskan, Asian, African American, Hispanic, Native Hawaiian/Pacific Islander, Caucasian). Functional status was measured using Activities of Daily Living (ADL) items: eating, bathing, locomotion, bed mobility, walking, toileting, transferring, hygiene, or dressing. A score ≥ 3 indicated difficulty with the ADL. Comorbidities at the advanced cognitive impairment incident assessment included indicators for diabetes mellitus, congestive heart failure, hypertension, chronic obstructive pulmonary disease (COPD), and stroke, as well as depression. Other health conditions included bowel or urinary incontinence (rarely or never vs. occasionally, frequently, or always), experiencing shortness of breath sitting or lying, pneumonia or other respiratory tract infection, urinary tract infection (UTI) in the previous 30 days, hip fracture in the prior 180 days, and weight-loss of more than 5 % in the last month or more than 10 % in the last 6 months.

### Statistical analysis

Descriptive analyses were conducted to identify differences in the predictors of survival between AD and ADRD residents with advanced cognitive illness using two-sided t-tests. For NH residents whose death date we observed in the follow-up period, survival was defined as the duration between their incident advanced disease assessment and date of death. Residents that survived until the end of the follow-up period were treated as censored. We estimated Cox multivariable survival models with nonparametric baseline survival functions stratified by sex for both AD and ADRD residents. We specified the models conditional on the predictor variables listed above and measured on the assessment identifying incident advanced cognitive impairment, as well as interactions between AD and ADRD and selected predictors. We assessed the proportional hazards assumption by examining log-log survival plots. Kaplan-Maier survival functions were developed separately by AD and ADRD as well as by sex. We calculated 25th, 50th and 75th percentiles in survival time (days) along with the probability of survival at the end of year 1, 2 and 3 since the onset of advanced cognitive impairment, both in total and by sex. All statistical analyses were performed using STATA MP, version 16 [34].

## Results

There were 34,493 (3.5 %) residents that met eligibility criteria for advanced cognitive impairment; among those, 35.0 % had AD and 65.0 % had ADRD. Most of the NH residents in our sample (i.e., with incident advanced cognitive impairment) died before the end of follow-up with only 11.9 % of the survival times censored.

Table 1 presents the comparison of AD and ADRD groups. About 65 % percent were female, and about 80 % were white non-Hispanic. All differences reported below are statistically significant at *p* < 0.01. At the onset of advanced cognitive impairment, NH residents diagnosed with ADRD had more comorbidities than those with AD, such as diabetes mellitus (28.88 and 24.28 %, respectively), stroke (21.57 and 12.48 %, respectively), and hypertension (74.83 and 71.12 %, respectively). NH residents with ADRD were also more likely to experience difficulties with ADLs, such as eating (66.35 and 63.76 %, respectively), walking (88.32 and 82.22 %, respectively), and locomotion (86.34 and 79.67 %, respectively), and to have experienced heart failure (19.44 and 15.16 %, respectively) and shortness of breath in the 7 days since the last MDS assessment (11.83 and 8.16 %, respectively). However, residents with ADRD were less likely than those with AD to experience depressive symptoms (41.52 and 45.64 %, respectively).

**Table 1:** Descriptive Statistics of NH residents with advanced cognitive impairment at the incident assessment

	AD (*N* = 12,093)		ADRD (*N* = 22,400)		Difference
	Mean (%)	(95% CI)	Mean (%)	(95% CI)	*p*-value
*Socio-demographics*					
Age: 65-74	10.51	(9.96, 11.06)	11.60	(11.18, 12.02)	-0.01**
Age: 75-84	38.34	(37.47, 39.2)	33.36	(32.74, 33.98)	0.05**
Age: 85-94	45.55	(44.66, 46.43)	46.38	(45.72, 47.03)	-0.01
Age: 95+	5.61	(5.2, 6.02)	8.66	(8.29, 9.03)	-0.03**
Married	36.01	(35.14, 36.87)	30.77	(30.16, 31.38)	0.05**
Caucasian	81.55	(80.86, 82.24)	77.42	(76.88, 77.97)	0.04**
Am. Ind. or Alaskan	0.20	(0.12, 0.28)	0.29	(0.22, 0.36)	-0.00
Asian	1.27	(1.07, 1.47)	2.16	(1.97, 2.35)	-0.01**
African American	9.76	(9.23, 10.29)	12.65	(12.21, 13.08)	-0.03**
Hispanic	5.56	(5.15, 5.97)	5.27	(4.97, 5.56)	0.00
Native Haw./Pac.Isl.	0.21	(0.13, 0.29)	0.39	(0.31, 0.47)	-0.00**
*Comorbidities*					
Type 2 Diabetes	24.28	(23.52, 25.05)	28.88	(28.29, 29.48)	-0.05**
Stroke	12.48	(11.89, 13.07)	21.57	(21.03, 22.11)	-0.09**
Hypertension	71.12	(70.32, 71.93)	74.83	(74.26, 75.4)	-0.04**
Depression	45.64	(44.75, 46.53)	41.52	(40.88, 42.17)	0.04**
Heart Failure	15.16	(14.52, 15.8)	19.44	(18.92, 19.96)	-0.04**
COPD	13.69	(13.07, 14.3)	16.80	(16.31, 17.29)	-0.03**
*Functional Status (ADL 3+)*					
*Difficulties with…*					
Walking	82.22	(81.54, 82.9)	88.32	(87.9, 88.74)	-0.06**
Eating	63.76	(62.91, 64.62)	66.35	(65.73, 66.97)	-0.03**
Bed mobility	86.58	(85.98, 87.19)	90.67	(90.29, 91.05)	-0.04**
Transfer	87.06	(86.46, 87.65)	91.67	(91.30, 92.03)	-0.05**
Locomotion	79.67	(78.96, 80.39)	86.34	(85.89, 86.79)	-0.07**
Dressing	94.07	(93.65, 94.49)	95.20	(94.92, 95.48)	-0.01**
Toilet	94.07	(93.65, 94.49)	95.65	(95.38, 95.91)	-0.02**
Hygiene	93.48	(93.04, 93.92)	94.48	(94.18, 94.78)	-0.01**
*Other Conditions*					
Bowel incontinence	87.68	(87.09, 88.27)	88.85	(88.43, 89.26)	-0.01**
Urinary incontinence	95.12	(94.71, 95.52)	95.22	(94.92, 95.53)	-0.00
Shortness of breath	8.16	(7.67, 8.65)	11.83	(11.4, 12.25)	-0.04**
Pneumonia	7.03	(6.57, 7.49)	9.00	(8.62, 9.37)	-0.02**
Urinary tract infect.	17.05	(16.38, 17.72)	19.45	(18.93, 19.97)	-0.02**
Hip fracture	6.29	(5.86, 6.73)	5.93	(5.62, 6.24)	0.00
Weight loss	17.38	(16.71, 18.06)	18.39	(17.89, 18.9)	-0.01*

*Note:* Mean and standard deviation reported for a list of covariates at the time of the incident advanced cognitive impairment assessment. *, and **, describe statistically significant difference between means at a *p*-value smaller or equal to 0.05 and 0.01, respectively, computed by conducting a two-sided equality of means t-test between advanced AD and ADRD groups

Abbreviations: *AD* Alzheimer’s disease, *ADRD* related dementias, *ADL* activities of daily living, *COPD* chronic obstructive pulmonary disease, *NH* nursing home

Table 2 presents the Cox survival regressions. Most predictors of death had similar hazard ratios (HR) for both AD and ADRD. In both AD and ADRD residents with advanced cognitive impairment, the following comorbidities were associated with greater mortality risk: diabetes mellitus (HR 1.11 and 1.12, respectively), heart failure (HR 1.24 and 1.26, respectively) and COPD (HR 1.13 for both). Mortality risks in AD and ADRD residents with advanced cognitive illness were highest for those who were experiencing shortness of breath (HR 1.65 and 1.54, respectively), substantial weight-loss (HR 1.31 and 1.35, respectively) or difficulty eating (HR 1.28 and 1.31, respectively) and pneumonia (HR 1.34 and 1.22, respectively). Difficulty with walking and bed mobility were both significant mortality risk factors for ADRD residents (HR 1.14 (*p* < 0.01) and 1.90 (*p* < 0.05), respectively) only. Experiencing stroke and difficulty with locomotion were both significant mortality risk factors for AD residents (HR 1.08 (*p* < 0.05) for both). Among the predictors, only having a stroke and being older than 95 years were significantly different between AD and ADRD residents.

**Table 2:** Mortality risk factor estimates using cox regression models for NH residents with advanced cognitive impairment

	*All (N = 29,058)*	
	AD Mean (95 % CI)	ADRD Mean (95 % CI)
*Socio-demographics*		
* Age: 75–84*	1.326** (1.229–1.432)	1.258** (1.186–1.335)
* Age: 85–94*	1.705** (1.579–1.841)	1.578** (1.488–1.674)
* Age: 95+*	2.158** (1.931–2.413)	1.840** (1.702–1.988)
* Married (= 1)*	1.135** (1.084–1.189)	1.128** (1.088–1.170)
* Am. Ind. or Alaskan*	1.278 (0.961–1.699)	0.900 (0.640–1.266)
* Asian*	0.746** (0.614–0.906)	0.608** (0.541–0.683)
* African American*	0.789** (0.731–0.852)	0.762** (0.722–0.804)
* Hispanic*	0.698** (0.631–0.772)	0.679** (0.629–0.732)
* Native Haw./Pac.Isl*	0.83 (0.567–1.216)	0.752* (0.571–0.990)
*Comorbidities*		
* Type 2 Diabetes*	1.107** (1.051–1.167)	1.122** (1.081–1.164)
* Stroke*	1.080* (1.012–1.152)	0.974 (0.935–1.014)
* Hypertension*	1.022 (0.974–1.073)	1.028 (0.990–1.068)
* Depression*	0.952* (0.912–0.994)	0.960* (0.929–0.992)
* Heart Failure*	1.240** (1.164–1.322)	1.262** (1.207–1.320)
* COPD*	1.133** (1.061–1.209)	1.135** (1.082–1.190)
*Functional Status (ADL 3+)*:		
Difficulties…		
* Walking*	1.053 (0.970–1.142)	1.143** (1.071–1.221)
* Eating*	1.277** (1.217–1.341)	1.313** (1.265–1.361)
* Bed mobility*	1.074 (0.982–1.175)	1.089* (1.005–1.180)
* Transfer*	1.106 (0.996–1.229)	1.025 (0.932–1.126)
* Locomotion*	1.086* (1.008–1.169)	1.056 (0.997–1.119)
* Dressing*	1.02 (0.902–1.153)	1.007 (0.915–1.108)
* Toilet*	0.97 (0.853–1.103)	1.036 (0.938–1.145)
* Hygiene*	1.032 (0.923–1.155)	0.988 (0.909–1.073)
* Other conditions*		
* Bowel incontinence*	1.071 (0.995–1.153)	1.048 (0.990–1.110)
* Urinary incontinence*	1.046 (0.934–1.172)	1.066 (0.976–1.163)
* Shortness of breath*	1.651*** (1.484–1.838)	1.539** (1.437–1.648)
* Pneumonia*	1.289** (1.157–1.437)	1.185** (1.105–1.271)
* Urinary Tract Infection*	1.024 (0.961–1.091)	1.096** (1.048–1.146)
* Hip fracture*	0.906* (0.828–0.992)	0.947 (0.882–1.016)
* Weight Loss*	1.311*** (1.230–1.397)	1.345** (1.285–1.408)

*Note*: *, and ** denotes coefficient significance at the 0.05, or 0.01 levels, respectively. Robust standard errors reported in brackets. Weight loss is recorded if the resident lost > 5 % of his or her weight last month or > 10 % in 6 months

Abbreviations: *AD* Alzheimer’s disease, *ADRD* related dementias, *ADL* activities of daily living, *COPD *chronic obstructive pulmonary disease, *NH* nursing home

There were differences in mortality risk factors for both AD and ADRD within sex (see Table 3). The largest mortality risk factors for women regardless of AD or ADRD diagnoses were shortness of breath, heart failure, difficulty eating, and weight-loss (HR 1.70, 1.26, 1.26, 1.31 for ACI-AD and HR 1.51, 1.27, 1.34 and 1.34, for ACI-ADRD; all *p* < 0.01). Stroke and difficulty with transfer were also significant mortality risk factors for women with AD (HR 1.16 and 1.20, respectively, both *p* < 0.01). Difficulty with walking (HR 1.17, *p* = 0.01), hypertension and urinary incontinence (HR 1.06 and 1.12, respectively, both *p* < 0.05) were significant mortality risk factors for women with ADRD. For men, diabetes mellitus, heart failure, difficulties with bed mobility or eating, and substantial weight-loss were significant risk factors regardless for both AD and ADRD residents (HR 1.10, 1.19, 1.37, 1.33, 1.21, 1.48, and 1.28, respectively, for AD; HR 1.12, 1.26, 1.31, 1.16, 1.16, 1.55, and 1.33, respectively for ADRD, all *p* < 0.05); and COPD was a significant risk factor for men with ADRD (HR 1.10, *p* < 0.05) but not for men with AD.

**Table 3:** Mortality risk factor estimates using cox regression models for NH residents with advanced cognitive impairment by sex

	*WOMEN (N = 19,575)*		*MEN (N = 9,483)*	
	**AD Mean (95 % CI)**	**ADRD Mean (95 % CI)**	**AD Mean (95 % CI)**	**ADRD Mean (95 % CI)**
***Socio-demographics***				
* Age: 75–84*	1.270** (1.147–1.406)	1.296** (1.192–1.409)	1.447** (1.288–1.627)	1.290** (1.184–1.406)
* Age: 85–94*	1.667** (1.507–1.843)	1.644** (1.514–1.784)	1.885** (1.668–2.129)	1.675** (1.533–1.831)
* Age: 95+*	2.170** (1.900–2.478)	1.966** (1.782–2.170)	2.486** (1.931–3.202)	1.994** (1.723–2.308)
* Married (= 1)*	0.999 (0.938–1.064)	0.976 (0.927–1.028)	0.981 (0.907–1.061)	1.042 (0.985–1.103)
* Am. Ind. or Alaskan*	1.525** (1.155–2.013)	1.082 (0.688–1.700)	0.89 (0.472–1.678)	0.654 (0.388–1.103)
* Asian*	0.82 (0.637–1.055)	0.642** (0.559–0.738)	0.601** (0.442–0.818)	0.550** (0.445–0.679)
* African American*	0.802** (0.728–0.882)	0.731** (0.682–0.783)	0.716** (0.627–0.816)	0.781** (0.717–0.851)
* Hispanic*	0.719** (0.633–0.816)	0.641** (0.579–0.709)	0.626** (0.530–0.739)	0.699** (0.624–0.784)
* Native Haw./Pac.Isl*	0.822 (0.540–1.252)	0.721 (0.518–1.004)	0.862 (0.370–2.006)	0.738 (0.455–1.197)
*Chronic conditions*				
* Type 2 Diabetes*	1.070* (1.001–1.144)	1.103** (1.053–1.156)	1.102* (1.013–1.200)	1.124** (1.055–1.197)
* Stroke*	1.159** (1.070–1.255)	0.984 (0.936–1.035)	0.916 (0.819–1.025)	0.932** (0.871–0.998)
* Hypertension*	1.037 (0.977–1.101)	1.060* (1.011–1.113)	1.013 (0.933–1.101)	0.998 (0.938–1.063)
* Depression*	0.974 (0.924–1.027)	0.991 (0.952–1.032)	0.968 (0.898–1.044)	0.950 (0.897–1.006)
* Heart Failure*	1.257** (1.164–1.358)	1.267** (1.199–1.337)	1.189** (1.062–1.332)	1.255** (1.160–1.359)
* COPD*	1.124** (1.035–1.220)	1.129** (1.064–1.199)	1.090 (0.977–1.216)	1.097* (1.016–1.185)
*Functional status (ADL 3+)*				
* Difficulties…*				
* Walking*	1.073 (0.971–1.186)	1.169** (1.077–1.269)	1.036 (0.902–1.189)	1.093 (0.984–1.214)
* Eating*	1.260** (1.188–1.338)	1.340** (1.281–1.402)	1.370** (1.259–1.491)	1.314** (1.234–1.399)
* Bed mobility*	0.974 (0.872–1.087)	1.073 (0.971–1.186)	1.331** (1.139–1.557)	1.163* (1.020–1.327)
* Transfer*	1.199** (1.051–1.367)	1.01 (0.897–1.137)	0.922 (0.768–1.107)	1.032 (0.884–1.205)
* Locomotion*	1.092 (0.997–1.195)	1.091* (1.017–1.172)	1.123 (0.987–1.278)	1.031 (0.937–1.134)
* Dressing*	0.981 (0.848–1.134)	1.015 (0.903–1.141)	1.082 (0.847–1.382)	1.004 (0.850–1.185)
* Toilet*	0.958 (0.822–1.116)	1.031 (0.910–1.167)	0.931 (0.725–1.196)	1.046 (0.887–1.233)
* Hygiene*	1.073 (0.937–1.230)	0.948 (0.857–1.049)	0.929 (0.757–1.140)	1.026 (0.887–1.187)
* Other conditions*				
* Bowel incontinence*	1.078 (0.986–1.179)	1.009 (0.941–1.082)	1.005 (0.886–1.141)	1.089 (0.986–1.202)
* Urinary incontinence*	1.062 (0.926–1.217)	1.121* (1.007–1.248)	1.132 (0.917–1.397)	0.986 (0.850–1.145)
* Shortness of breath*	1.699** (1.480–1.949)	1.509** (1.382–1.647)	1.481** (1.245–1.762)	1.553** (1.392–1.732)
* Pneumonia*	1.259** (1.088–1.457)	1.108* (1.006–1.221)	1.210* (1.027–1.426)	1.163** (1.052–1.285)
* Urinary Tract Infection*	1.051 (0.977–1.131)	1.119** (1.062–1.179)	1.1 (0.965–1.254)	1.170** (1.071–1.277)
* Hip fracture*	0.961 (0.864–1.068)	0.945 (0.870–1.026)	0.797* (0.665–0.956)	1.034 (0.901–1.187)
* Weight Loss*	1.313** (1.216–1.417)	1.340** (1.268–1.416)	1.281** (1.143–1.437)	1.331** (1.227–1.444)

*Note*: *, and ** denotes coefficient significance at the 0.05, or 0.01 levels, respectively. Robust standard errors reported in brackets. Weight loss is recorded if the resident lost > 5 % of his or her weight last month or > 10 % in 6 months

Abbreviations: *AD* Alzheimer’s disease, *ADRD* related dementias, *ADL* activities of daily living, *COPD* chronic obstructive pulmonary disease, *NH* nursing home

**Fig. 1:**
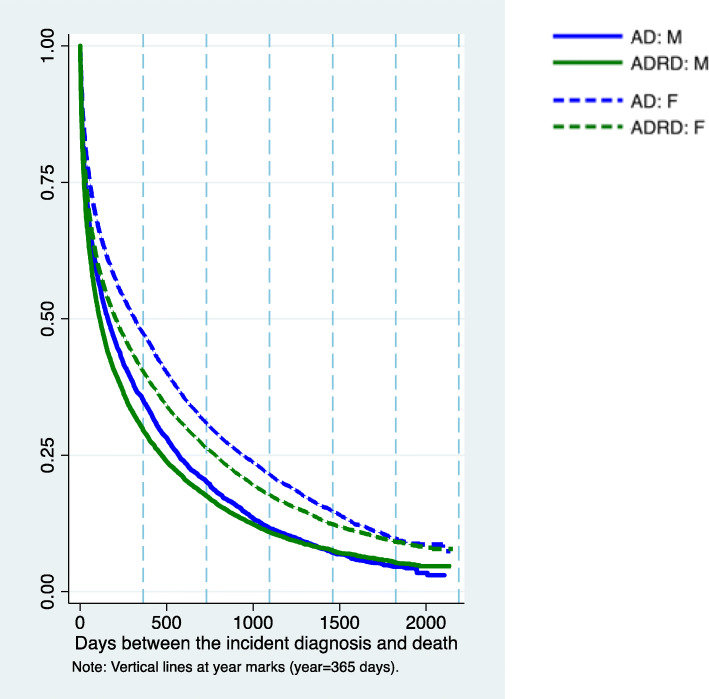
Survival functions of nursing home residents with advanced cognitive impairment by diagnosis type and sex. *Note*: Author’s estimation of the Kaplan-Meier survivor function. Abbreviations: AD, Alzheimer’s disease; ADRD, related dementias; F, female; M, male

As presented in Fig. 1; Table 4, the median survival times were on average 300 (CI95 % 286 to 315) and 194 (CI95 % 186 to 202) days for AD and ADRD residents, respectively. By the end of year 1, the probability of survival for NH residents was 46.3 % (CI95 % 45.4 to 47.2), and 39.0 % (CI95 % 39.4 to 39.7) for those with AD and ADRD, respectively. Absolute differences in survival rates between disease groups decreased with time, and by year 3 the rate of survival for NH residents with AD was 19.9 % (CI95 % 19.2 to 20.7) compared to 16.6 % (CI95 % 16.1 to 17.1) for those with ADRD. Men had substantially lower survival rates than women throughout the first three years regardless of the dementia diagnosis. For instance, by year 3, the survival rate for women with AD was 23.5 % (CI95 % 22.6 to 24.4) compared to 19.2 % (CI95 % 18.6 to 19.9) for those with ADRD. For men, the survival rate at year 3 was around 12 % regardless of dementia diagnosis.

**Table 4:** Survival probability and time since incident advanced cognitive impairment and death

		Survival time in days (CI 95 %)			Probability of survival (%)			
	**N**	**25 % (CI 95 %)**	**50 % (CI 95 %)**	**75 % (CI 95 %)**		**Year 1 (CI 95 %)**	**Year 2 (CI 95 %)**	**Year 3 (CI 95 %)**
AD	11,978	57 (54, 62)	300 (286, 315)	891 (856, 920)		46.3 (45.4, 47.2)	29.6 (28.8, 30.5)	20.0 (19.2, 20.7)
*Women*	8,087	74 (67, 78)	374 (352, 395)	1031 (996, 1069)		50.4 (49.3, 51.5)	33.4 (32.4, 34.4)	23.5 (22.6, 24.4)
*Men*	3,891	38 (35, 43)	192 (177, 209)	623 (583, 661)		38.0 (36.4, 39.5)	21.8 (20.5, 23.1)	12.6 (11.6, 13.7)
ADRD	22,045	35 (33, 36)	194 (186, 202)	731 (712, 752)		39.0 (39.4, 39.7)	25.0 (24.4, 25.6)	16.6 (16.1, 17.1)
*Women*	14,296	41 (39, 44)	246 (233, 260)	845 (820, 871)		43.0 (42.2, 43.8)	28.4 (27.6, 29.1)	19.2 (18.6, 19.9)
*Men*	7,749	27 (25, 29)	132 (124, 140)	517 (494, 550)		31.7 (30.7, 32.7)	18.8 (18.0, 19.7)	11.7 (11.0, 12.5)

*Note*: 25th, 50th, and 75th percentile of survival times is obtained from S(t), the Kaplan-Meier estimate of the survivor function in Fig. 1. Survival time is reported in days. We report 95 % confidence intervals in the brackets

Abbreviations: *AD* Alzheimer’s disease, *ADRD* related dementias, *CI* confidence interval

## Discussion

Significant differences were found in health status at the incident advanced cognitive impairment, as well as in survival patterns. We find that among NH residents with advanced cognitive impairment, on average, those with ADRD are of worse health compared to those with AD at their incident assessment of advanced cognitive impairment as observed in MDS. This may be largely because ADRD is a heterogenous collection of conditions that likely have different underlying causes, co-morbidities and the rate of disease progression, but it may also be that ADRD is diagnosed later in life than AD, particularly in a NH environment [13, 14, 20, 23]. On average, residents with ADRD are more likely than those with AD to be older than 85 at the incident advanced cognitive impairment assessment.

On the other hand, most of mortality risk factors were similar across groups. This highlights an important fact which is that regardless of the type of dementia, issues that residents, as well as families and caregivers are confronted with at the end of one’s life are similar. For both, AD and ADRD residents with advanced cognitive impairment, the largest mortality risk factors were shortness of breath, pneumonia, weight loss and difficulties with eating; and these were similar for both, men and women. Stroke and difficulty with locomotion were a significant risk factor for women with AD only, but difficulty with bed mobility, walking and UTI were significant risk factors for residents with ADRD, regardless of sex. These results are consistent with the literature. For instance, eating difficulties and related weight-loss are common complications in patients with AD, and are a significant predictor of end of life [14, 17]. On the other hand, for patients with ADRD such as DLB, walking and fall-related injuries were among the largest mortality risks, possibly because these patients displayed severe deficits in attention and visuospatial processing, whereas these were not as a substantial risk factors for patients with AD. Patients with Parkinson’s disease, for instance, have been found to have increased risk for UTI due to voiding dysfunctions, but also more commonly experienced swallowing disorders leading to eating difficulties and significant weight-loss. An important difference that we identified between AD and ADRD with advanced disease compared to the existing evidence is that stroke was mainly a significant risk factor for residents with AD, especially women, and not for those with ADRD. Other evidence shows that for some other types of ADRD, such as vascular for dementia, hypertension, diabetes, and stroke remain the most important mortality risk factors [20, 35].

We also observe that mortality risk was consistently higher for whites (vs. non-whites) among residents diagnosed with either advanced AD or ADRD. Consistent with prior work, these results could be driven by a sample selection due to lower institutionalization rates among non-white residents with AD or ADRD [36]. Thus, non-white residents in our sample may have been wealthier and/or of better health than the white NH residents or average non-whites in community who we do not observe. There may be several unobserved mediators in our data that could explain the differences in mortality risk, such as genetics, residents’ socio-economic status, available resources, social networks, and social capital, as well as unobserved psychological stressors, discrimination, or access to medical or home or other care [37, 38].

Our results regarding survival are also supported by published literature. Researchers focusing on *severe* dementia (either AD or ADRD) have found that the median survival ranges from 2 days to 16 months but variability in survival time estimates is high [29, 31, 32]. Our survival rate estimates are similar to those found by Mitchell et al. 2010, who found that about 40 % of patients with advanced dementia (either AD or ADRD) die within 1 year [29]. We add to this evidence by showing that there are differences in expected survival for those with AD compared to those with ADRD as identified in MDS, particularly in the first year after their onset of advanced cognitive impairment in which those with ADRD have higher mortality rates. Specifically, our estimated survival rate at year 1 is 46.3 % for AD and 39 % for ADRD residents (with 65 % having ADRD). Like us, other investigators have found that the risk of mortality is smaller for patients with AD than for those with the ADRD but these studies have typically not limited their focus to those with advanced cognitive impairment and did not use MDS to differentiate the groups [11, 33]. As others, we find lower survivals for men in both groups [11, 21, 29].

This study has limitations. First, diagnostics of the advanced cognitive impairment may be imprecise or under reported, particularly given the difficulty in diagnosing AD and ADRD. Though some of the advanced cognitive impairment cases may be missed [39], MDS data includes strict protocols about data reporting as well as the timing of assessments, and MDS 3.0 in particular has shown either excellent or very good reliability in assessments, their clinical relevance, and low rates of missing responses [40]; largely addressing concerns that the diagnoses are misclassified. Many have used similar measures to ours to identify cognitive impairments [14, 27, 29, 41, 42]. Second, ADRD arises from a variety of heterogeneous conditions, only some of which are identifiable (and imperfectly at that) in the MDS data. The extent to which each of those conditions gives rise to different patterns of survival in our data remains unknown, but the heterogeneity of ADRD may explain the different patterns of survival between AD and ADRD. Additionally, we cannot identify residents with a specific condition (e.g., DLB only) or exclude those usually not included in the ADRD definition (e.g., Creutzfeldt-Jakob disease). Third, an important goal of this paper was to define measures of advanced cognitive impairment that would be highly predictive of mortality, and although a large fraction of those identified with advanced disease died relatively quickly, the survival functions still have a long tail. Fourth, whether the first observed incident of advanced cognitive impairment in MDS is the actual time advanced disease is identified is unknown yet unavoidable due to data limitations. Because advanced cognitive impairment definitions require assessment data (complete with data on physical and cognitive limitations) we can only identify the first time the cognitive decline is observed in MDS for those who resided in NH before the disease progressed to advanced illness. As a result, we could identify the entire period of advanced cognitive impairment for only a fraction of those who reside in NH. Though other clinical data may provide a more precise measure of the advanced cognitive impairment onset, our study utilizes MDS, which is a relatively easily accessible data source for other researchers. Since our focus is end of life, identifying the incident advanced cognitive impairment diagnosis as observed in the MDS is informative in that it provides a conservative estimate of survival for people with advanced disease once admitted to NH. Finally, though we studied mortality risk factors, our data did not allow us to investigate pathways or mediators giving rise to these factors, such as genetics, residents’ socio-economic status, resources, social network or ties, as well as psychological stressors, discrimination, access to care or idiosyncratic behaviors [37, 38]. These questions are important to address in future research.

## Conclusions

This study provided new evidence showing that routine MDS assessments may be used to differentiate between mortality risks and survival between AD and ADRD overall and by sex. Though mortality risks for NH residents with advanced ADRD and AD were largely similar, those with ADRD had worse health status on average and shorted survival from their observed incident of advanced cognitive impairment. Findings may inform future developments of risk tools used to identify end of life among AD and ADRD residents and help inform care planning decisions regarding therapeutic and palliative care.

## Supplementary Information

**Additional file 1: eFigure 1: **Survival and hazard function comparison for those diagnosed with advanced ADRD, AD, or both.

## Data Availability

The data that support the findings of this study are available from the Centers of Medicare and Medicaid but restrictions apply to the availability of these data, which were used under license for the current study, and so are not publicly available. Data are however available from the authors upon reasonable request and with permission of Centers of Medicare and Medicaid.

## Abbreviations

AD: Alzheimer’s disease
ADL: activities of daily living
ADRD: Alzheimer’s related dementia
CFS: Cognitive function score
COPD: Chronic obstructive pulmonary disease
MDS: Minimum Data Set
NH: Nursing home
UTI: Urinary tract infections
