# Supplementary material for: Advanced cognitive impairment among older nursing home residents
Source: BMC Geriatr. 2021 Jun 23;21:382. doi: 10.1186/s12877-021-02336-1 (PMC8220753; doi:10.1186/s12877-021-02336-1)
Supplement: Supplementary file 1 — Additional file 1: eFigure 1: Survival and hazard function comparison for those diagnosed with advanced ADRD, AD, or both. [file 12877_2021_2336_MOESM1_ESM.docx]

**eSUPPLEMENT**

***Title:* Advanced cognitive impairment among older nursing home residents**

**Authors:** Tadeja Gracner, Patricia W. Stone, Mansi Agarwal, Mark Sorbero, Susan L Mitchell, Andrew W. Dick

**Content:**

**eFigure 1:** Survival and hazard function comparison for those diagnosed with advanced ADRD, AD, or both.

**eSupplement 1**

**eFigure 1:** Survival and hazard function comparison for those diagnosed with advanced ADRD, AD, or both.

1. Kaplan-Meier Survival Function

**
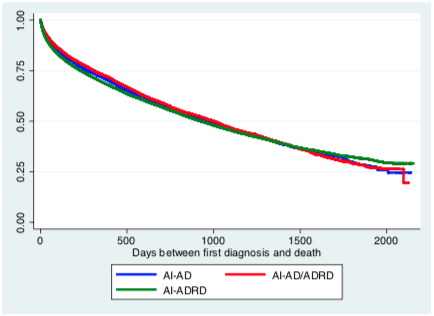
**

Advanced AD and ADRD

Advanced ADRD

Advanced AD

1. Hazard function

**
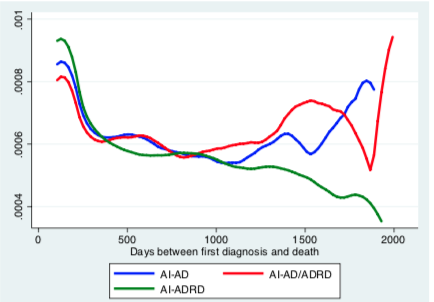
**

*Note*: Author’s estimation of the Kaplan-Meier survivor and hazard function for each mutually exclusive group: NH residents with advanced AD, ADRD or AD and DRD. Abbreviations: AD, Alzheimer’s disease; ADRD, related dementias.
